# Clinical significance and immune characteristics analysis of miR-221-3p and its key target genes related to epithelial-mesenchymal transition in breast cancer

**DOI:** 10.18632/aging.205370

**Published:** 2024-01-06

**Authors:** Yutong Fang, Qunchen Zhang, Zexiao Chen, Cuiping Guo, Jundong Wu

**Affiliations:** 1 The Breast Center, Cancer Hospital of Shantou University Medical College, Shantou 515041, Guangdong, China; 2 Department of Central Laboratory, Cancer Hospital of Shantou University Medical College, Shantou 515041, Guangdong, China; 3 Department of Breast, Jiangmen Central Hospital, Jiangmen 529000, Guangdong, China

**Keywords:** breast cancer, miR-221-3p, epithelial-mesenchymal transition, target gene, diagnosis, prognosis, immune infiltration, drug sensitivity

## Abstract

Background: MicroRNA-221-3p (miR-221-3p) facilitates the advancement of breast cancer (BC) through the induction of epithelial-mesenchymal transition (EMT). Our research aimed to utilize bioinformatics to discover possible EMT-related target genes (ETGs) of miR-221-3p and examine their roles in breast cancer.

Methods: We employed bioinformatics techniques to identify ten key ETGs of miR-221-3p. Subsequently, we conducted an extensive analysis of both miR-221-3p and the ten ETGs, including clinical significance and immune characteristics.

Results: The expression of miR-221-3p was notably higher in Basal-like BC compared to other subtypes and adjacent normal tissue. Our pathway analysis suggested that miR-221-3p might regulate EMT through the MAPK signaling pathway by targeting its ETGs. Among the ETGs, seven core genes (EGFR, IGF1, KDR, FGF2, KIT, FGFR1, and FGF1) exhibited downregulation in BC. Conversely, ERBB2, SDC1, and MMP14 showed upregulation in BC and displayed potential diagnostic value. The analysis of prognostication indicated that increased levels of SDC1 and MMP14 were correlated with an unfavorable prognosis, whereas elevated expression of KIT was associated with a more favorable prognosis. The infiltration of various immune cells and the expression of immune checkpoint genes (ICGs) exhibited positive correlations with most ETGs and miR-221-3p. SDC1 exhibited a greater tumor mutational burden (TMB) score, while ERBB2, KDR, FGF2, KIT, FGFR1, and FGF1 showed lower TMB scores. Furthermore, decreased ERBB2 and KDR expression levels were correlated with elevated microsatellite instability (MSI) scores. Elevated expression of ETGs was linked to decreased mRNA stemness indices (mRNAsi), whereas miR-221-3p displayed the opposite pattern. Most ETGs and miR-221-3p expression exhibited a negative correlation with IC50 values for drugs. Among the ETGs, amplification was the most significant genetic alteration, except for IGF1.

Conclusion: In conclusion, miR-221-3p acts as a unique indicator for Basal-like BC. The examination revealed ten essential ETGs of miR-221-3p, some of which show potential as diagnostic and prognostic markers. The in-depth examination of these ten ETGs and miR-221-3p indicates their participation in the development of BC, emphasizing their promise as innovative targets for therapy in BC patients.

## INTRODUCTION

Breast cancer (BC) presents a major challenge for women worldwide due to its high incidence rate, with over two million new cases diagnosed annually [1]. A Comprehensive therapeutic strategy is currently used in the treatment of BC patients [2]. Patients with localized BC have a survival rate of over 90% within five years, whereas those with metastatic cancer experience a rate below 30% [3]. Metastasis is the primary reason for more than 90% of cancer-related fatalities in BC [4]. Tumor metastasis is closely linked to the crucial process of epithelial-mesenchymal transition (EMT), where epithelial cells undergo a change into highly mobile mesenchymal cells that possess increased migratory and invasive abilities [5]. The process of epithelial-mesenchymal transition (EMT) has been linked to multiple biological aspects of breast cancer (BC), such as the development of stem cell-like traits [6]. Besides, the process of EMT has been recognized as a crucial element in the immunosuppression within the microenvironment of the tumor, facilitating cancer progression and contributing to drug resistance [7]. Furthermore, substantial evidence suggests that EMT-related genes are linked to therapeutic resistance. Hence, it is crucial to evaluate the influence of gene expression related to EMT in order to develop accurate and individualized therapeutic approaches for BC patients [8].

MicroRNAs (miRNAs) are small RNA molecules that are not involved in coding and have a significant function in regulating gene expression after transcription. Which involved the development and advancement of different human illnesses, such as cancers [9, 10]. Several studies have revealed that miRNAs can trigger the EMT process to promote tumor invasion and metastasis in various malignancies [11]. Furthermore, specific miRNAs related to EMT have been discovered to be linked to the characteristics of cancer stem cells and resistance to drugs [12].

MiRNA-221-3p belongs to the miRNA family that is encoded on the X chromosome in humans. The regulation of its target genes is essential in the growth of different cancerous tumors, as it can either inhibit or facilitate tumorigenesis. [13]. Previous research has provided evidence that miR-221-3p exerts regulatory control over the expression of its target genes, thereby regulating the process of EMT in BC [14, 15]. Considering that miRNAs can regulate multiple targets simultaneously [16], further exploration of the underlying molecular mechanisms through which miR-221-3p regulates the EMT process in BC is warranted.

Considering the information mentioned, our research aimed to discover further possible target genes linked to EMT that are regulated by miR-221-3p. Moreover, our aim was to investigate their clinical importance, their correlation with immune cells that infiltrate tumors, and their influence on drug responsiveness through the utilization of bioinformatics tools. The discovery of these results has the capacity to reveal new targets for therapy in individuals with BC.

## MATERIALS AND METHODS

### MiR-221-3p differential expression analysis

Clinical information for BC patients was obtained along with the miR-221-3p expression data from the Cancer Genome Atlas (TCGA) website, which included 1103 BC tissue and 104 normal samples. The expression data of miR-221-3p was displayed in log2(RPM+1) format. Furthermore, we verified the distinct expression of miR-221-3p by utilizing the GSE45666 dataset obtained from the Gene Expression Omnibus (GEO) database and various cell lines. Receiver Operating Characteristic (ROC) curve analysis was performed to assess the discriminatory power of miR-221-3p in distinguishing BC subtypes.

### EMT-related target genes (ETGs) identification and enrichment analysis

In order to discover possible target genes of miR-221-3p (Supplementary Table 1), we utilized version 3.0 of miRWalk, a website that integrates prediction results from multiple databases for comprehensive target gene analysis [17]. From the dbEMT 2.0 database [18], a collection of 1184 genes associated with EMT (Supplementary Table 2) was acquired [18]. Furthermore, the R software package limma was used to identify the differentially expressed genes (DEGs) (Supplementary Table 3) using data from TCGA. The criteria of |logFC| > 1 and FDR < 0.05 were applied as thresholds. Subsequently, we pinpointed the common genes shared among the possible target genes of miR-221-3p, the DEGs, and the EMT-related genes, thereby designating them as the possible ETGs of miR-221-3p. For a deeper investigation into the pathways associated with BC progression and the biological roles of the potential ETGs of miR-221-3p, we conducted Gene Ontology (GO) and Kyoto Encyclopedia of Genes and Genomes (KEGG) functional enrichment analyses, which were executed with the R clusterProfiler package. To improve the accuracy of the research, we created a network of protein-protein interactions (PPI) for the ETGs by utilizing the STRING database [19]. The visualization of the network was done using Cytoscape version 3.8.2. For further investigation, the top ten core genes were selected as the ETGs of miR-221-3p using the cytoHubba tool.

### Correlation analysis

The RNA-sequencing data for ten ETGs were obtained from the TCGA website. Subsequently, the data were transformed into transcripts per million (TPM) format, represented as log2(TPM+1). Then we analyzed the relation between miR-221-3p and its ETGs, as well as the pairwise relation among the ETGs.

### ETGs differential expression analysis

Using data obtained from TCGA, we analyzed the differential expression of ten ETGs between the normal and BC groups. The validation of this analysis was later confirmed by the GSE45666 dataset of the GEO database and cell lines. Additionally, we obtained immunohistochemistry (IHC) images of BC tissues and normal tissues of ten ETGs from the Human Protein Atlas (https://www.proteinatlas.org/) website for further analyzing their expression at the protein level.

### Quantitative real-time PCR (qRT-PCR) analysis

The BC cell lines MCF-7 and MDA-MB-231, as well as the breast epithelial cell line MCF-10A, were procured from Procell (Wuhan, China) and cultured following the manufacturer’s guidelines. Total RNA was extracted from the cells using the RNAsimple total RNA kit (Tiangen, Beijing, China) according to the manufacturer’s instructions. Subsequently, qRT-PCR was performed using the PrimeScript^™^ RT reagent kit (Takara, Japan) and the SYBR Premix Ex Taq^™^ II (Takara, Japan) as per the manufacturer’s protocol. GAPDH was chosen as the internal reference gene, and the relative expression levels were calculated using the 2^−ΔΔCt^ method. Specific primers used in this study are shown in Table 1.

**Table 1:** Sequences of all Primers.

	**Primers sequence (5′–3′)**
GAPDH F	GTCAAGGCTGAGAACGGGAA
GAPDH R	TGGACTCCACGACGTACTCA
EGFR F	TCAGCTAGTTAGGAGCCCATTTTT
EGFR R	TGTGACTGAACATAACTGTAGGCT
IGF1 F	TCTCTAAATCCCTCTTCTGTTTGCT
IGF1 R	GGAGATGTTGAGAGCAATGTCAC
ERBB2 F	TCTGCTGGCATCAAGAGGTG
ERBB2 R	AGCCATCTGGGAACTCAAGC
KDR F	GTTCAGACGGGGTTTCTGGT
KDR R	TTGGCCAGGAGACACGTAAC
FGF2 F	GTGCTAACCGTTACCTGGCT
FGF2 R	TCTGCCCAGGTCCTGTTTTG
KIT F	AGGTTGTTGAGGCAACTGCT
KIT R	ATGGTGCAGGCTCCAAGTAG
FGFR1 F	GAGCCTTGTCACCAACCTCT
FGFR1 R	AAGCATCTCACCGAAATCCCG
SDC1 F	GGAAGGGCCTGTGGGTTTAT
SDC1 R	CTGCTCGATGCTCTCTTGGG
FGF1 F	CGGCTCAACACCAAATGAGG
FGF1 R	TCTGGCCATAGTGAGTCCGA
MMP14 F	CCGATGTGGTGTTCCAGACA
MMP14 R	TCGTATGTGGCATACTCGCC
MiR-221-3p F	GTTCGTGGGAGCTACATTGTCTGC
MiR-221-3p R	GTGTCGTGGAGTCGGCAATTC
MiR-221-3p RT Primer	GTCGTATCCAGTGCAGGGTCCGAGGTATTCGCACTGGATACGACGAAACCCA

### IHC staining and scoring

We used tissue microarrays (F048Br01a, Bioaitech, China) comprising 24 BC tissues of varying stages and grades, along with their corresponding adjacent tumor tissues. IHC staining and scoring were conducted following a previously described protocol [20]. In summary, following the removal of paraffin, rehydration, and microwave antigen retrieval, the slides were left to incubate with antibodies overnight at a temperature of 4 degrees Celsius. Anti-ERBB2 (ab237715, Abcam), anti-SDC1 (ab130405, Abcam), and anti-MMP14 (ab51074, Abcam) were each diluted to concentrations of 1:2000, 1:1000, and 1:800, respectively. Afterward, the slides were subjected to secondary antibodies at ambient temperature for a duration of 30 minutes. Then, they were stained using DAB substrate and subsequently counterstained with hematoxylin.

### ETGs clinical significance analysis

The diagnostic value of the upregulated ETGs was evaluated through ROC curve analysis, and the findings were subsequently confirmed using the GSE45666 dataset. To assess the clinical significance of the ETGs, we conducted an analysis of their expression in relation to clinical stages and PAM50 subtypes of BC. Furthermore, to investigate the correlation between the ETGs expression and the prognosis, we stratified the BC patients into two groups according to the median ETGs expression value. Subsequently, the Kaplan-Meier (KM) survival analysis was conducted to analyze the relationship between ETGs expression and patient prognosis, encompassing both overall survival (OS) and the disease-specific survival (DSS).

### Immune characteristics analysis

To investigate the correlation between ETG expression and immune cells, we employed the single-sample gene set enrichment analysis method [21] to evaluate the infiltration enrichment of 24 common immune cell types. This enabled us to conduct further correlation analysis between ETGs expression and immune cell infiltration levels. Furthermore, the correlation between eight immune checkpoint genes (ICGs) and ETGs expression was analyzed.

### Tumor mutational burden (TMB), microsatellite instability (MSI) and stemness analysis

TMB and MSI scores were derived from prior research [22, 23]. The one-class logistic regression machine-learning algorithm [24] facilitated the computation of the mRNA expression-based stemness index (mRNAsi) score. Analysis of the difference of the TMB, MSI and mRNAsi scores between the high and low expression groups of ten ETGs and miR-221-3p was conducted.

### Drug sensitivity analysis

To predict the drug response of individual samples obtained from TCGA, we employed the R pRRophetic package. Afterward, the IC50 values for each sample’s drug sensitivity were estimated using Ridge’s regression, utilizing data acquired from the Genomics of Drug Sensitivity in Cancer database [25]. Additionally, we conducted further analysis on the association between the IC50 values and the expression of the ETGs and miR-221-3p.

### Genetic alteration analysis

We utilized the cBioPortal website to analyze genetic alterations in the BC cohort. Moreover, we examined OS and DSS in both the altered and unaltered groups.

### Statistical analysis

The unpaired *t*-test was used to analyze statistical differences between two groups, whereas the Kruskal-Wallis test was employed for comparing multiple groups. Expression level values were presented as means ± standard deviations. Correlation analysis utilized Spearman’s correlation test. Statistical analyses and plotting were made easier with the help of R software (version 4.2.1) and GraphPad Prism (version 8.0). Statistically significant findings were determined in this study for *P*-values less than 0.05.

### Availability of data and materials

The data underlying this study are freely available from TCGA (https://portal.gdc.cancer.gov/), GEO (http://www.ncbi.nlm.nih.gov/geo/), miRWalk 3.0 (http://mirwalk.umm.uni-heidelberg.de/), dbEMT 2.0 (http://dbemt.bioinfo-minzhao.org/index.html), Human Protein Atlas (https://www.proteinatlas.org/) and cBioPortal databases (http://www.cbioportal.org).

## RESULTS

### The clinical significance and differential expression of miR-221-3p

**Figure 1:**
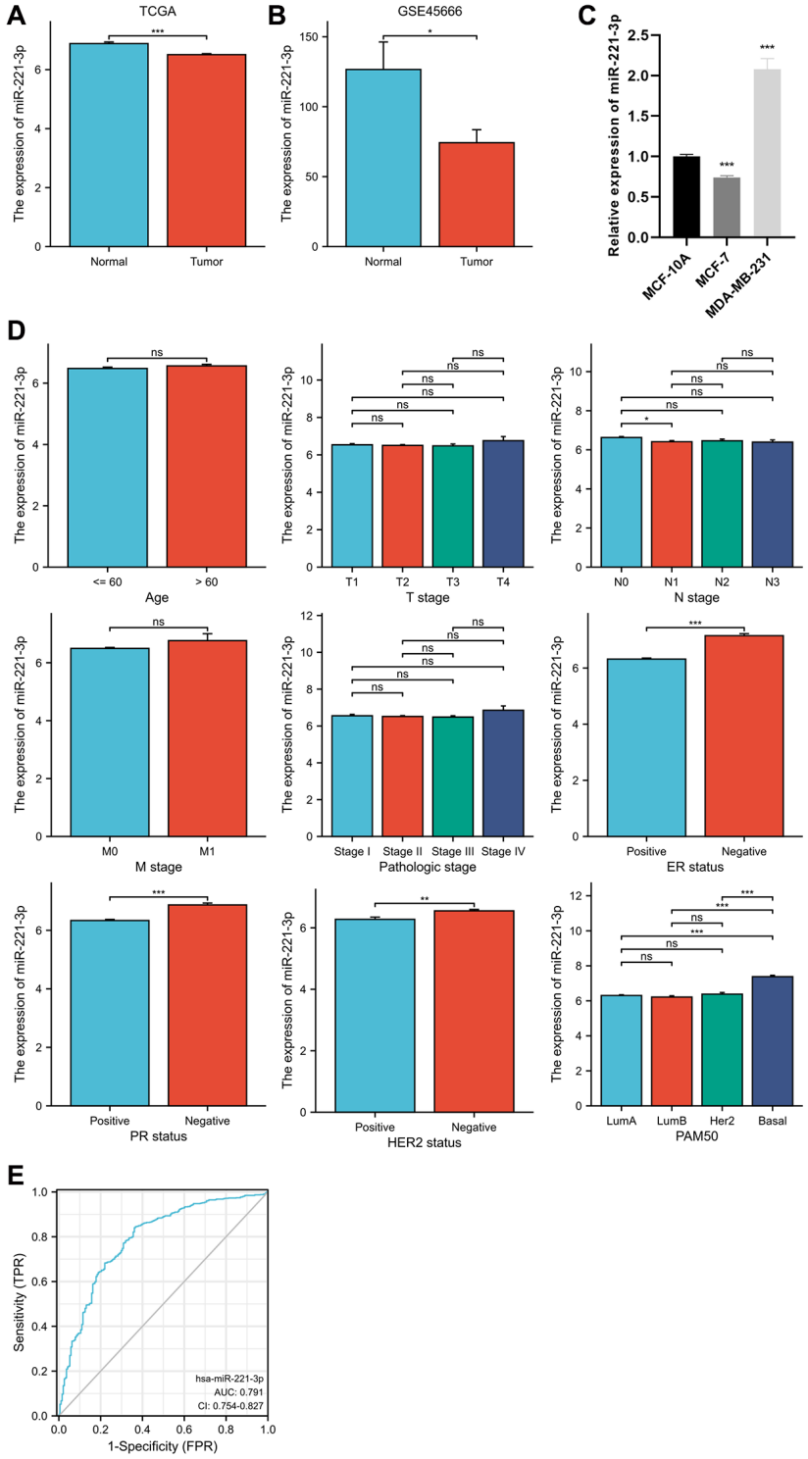
MiR-221-3p differential expression in BC and normal adjacent tissues based on TCGA database (**A**) and validated by the GSE45666 dataset (**B**) and cell lines (**C**). The clinical significance of miR-221-3p expression (**D**). ROC curve shows the discriminative power of miR-221-3p between the Basal-like subtype and others (**E**). In the present study, NS indicates no statistical difference, ^*^P < 0.05, ^**^P < 0.01, ^***^P < 0.001, ^****^P < 0.0001.

The differential expression analysis of miR-221-3p revealed that miR-221-3p levels were lower in BC tissues compared to normal tissues (*p* = 0.001). In Figure 1A, the expression levels in BC tissues and normal tissues were 6.509 ± 1.082 and 6.884 ± 0.557, respectively. The GSE45666 dataset also confirmed the identical outcome (*p* < 0.05) (Figure 1B). Moreover, miR-221-3p expression was decreased in MCF-7 and notably increased in MDA-MB-231 (Figure 1C). Furthermore, as shown in Figure 1D, the higher expression of miR-221-3p was linked to the negative status of estrogen receptor (ER) (*p* < 0.001) and progesterone receptor (PR) (*p* < 0.001), as well as human epidermal growth factor receptor 2 (HER2) (*p* = 0.004). Additionally, miR-221-3p expression was found to be reduced in BC patients with nodal status N1 in comparison to individuals without lymph node metastases (*p* = 0.044). Significantly, miR-221-3p expression in the Basal-like subtype was 7.379 ± 1.081, markedly higher than that in Luminal A (6.303 ± 0.975, *p* < 0.001), Luminal B (6.214 ± 1.013, *p* < 0.001), and HER2-enriched subtypes (6.380 ± 0.845, *p* < 0.001). Due to the high expression in Basal-like BC, we further prove miR-221-3p is a special biomarker of which to discriminate from other subtypes. Using a cut-off value of 7.037, the ROC curve analysis yielded an area under the curve (AUC) of 0.791, with a specificity of 63.9% and sensitivity of 84.0% (Figure 1E).

### ETGs identification and enrichment analysis

**Figure 2:**
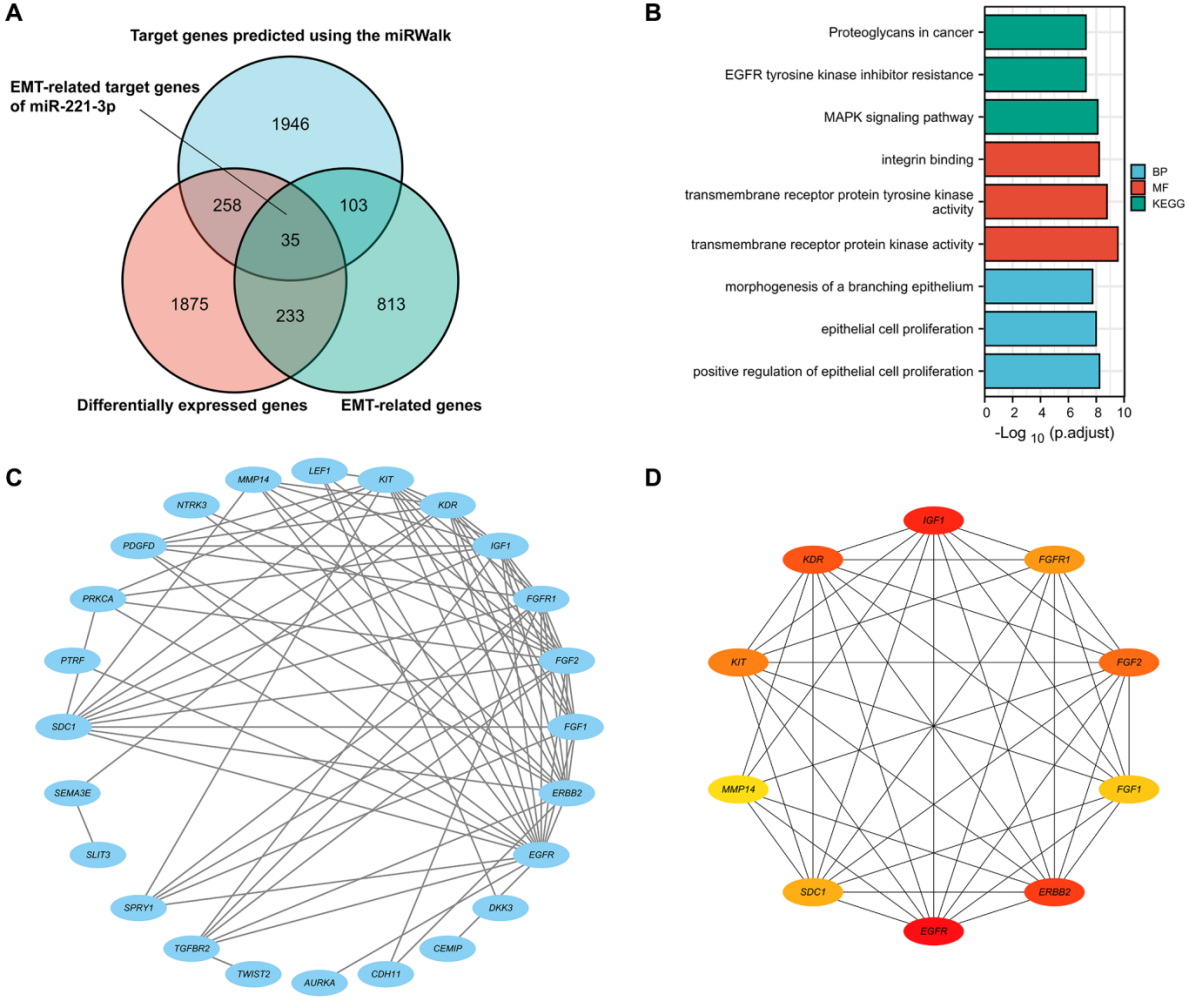
The Venn diagram shows DEGs, EMT-related genes, and possible targets of miR-222-3p (**A**). GO and KEGG pathway enrichment analysis of 35 ETGs (**B**). The PPI network of 35 ETGs (**C**). 10 top core genes of the PPI network were identified as the ETGs of miR-221-3p for further research (**D**).

As shown in the Venn diagram (Figure 2A), 35 genes were selected as possible ETGs of miR-221-3p. The subsequent GO analysis of these 35 genes revealed that they may play a role in regulating biological processes related to epithelial cell proliferation and transmembrane receptor protein tyrosine kinase activity. Furthermore, the KEGG pathway analysis suggested that these ETGs might play a role in controlling the EMT process via the MAPK signaling pathway and could potentially be linked to the resistance of EGFR tyrosine kinase inhibitors in the BC treatment (Figure 2B; Supplementary Table 4). Figure 2C shows the construction of a PPI network of 35 ETGs, including 26 nodes and 78 edges. In order to enhance the precision of predictions, we have identified the top ten core genes from the PPI network for further investigation. These core genes included EGFR, IGF1, ERBB2, KDR, FGF2, KIT, FGFR1, SDC1, FGF1, and MMP14 (Figure 2D).

### Correlation analysis

**Figure 3:**
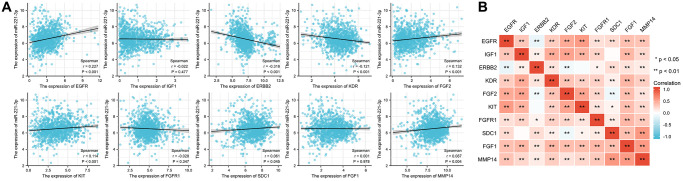
The correlation between miR-221-3p and its ETGs expression (**A**). The pairwise correlation among the ETGs expression (**B**).

Correlation analysis revealed that seven out of the ten ETGs exhibited a significant correlation with miR-221-3p expression. The results depicted in Figure 3A indicate that the levels of EGFR (r = 0.227, *p* < 0.001), FGF2 (r = 0.132, *p* < 0.001), KIT (r = 0.114, *p* < 0.001), SDC1 (r = 0.061, *p* = 0.045), and MMP14 (r = 0.087, *p* = 0.004) exhibited a positive correlation with miR-221-3p expression. In contrast, miR-221-3p expression showed a negative correlation with ERBB2 (r = −0.318, *p* < 0.001) and KDR (r = −0.121, *p* < 0.001). Moreover, the ETGs were evaluated for pairwise correlation, and the majority of them exhibited positive correlation with one another (Figure 3B). Additionally, it was observed that there were negative correlations between EGFR and ERBB2 (r = −0.142, *p* < 0.001), FGF2 and ERBB2 (r = −0.088, *p* = 0.003), and FGF2 and SDC1 (r = −0.108, *p* < 0.001).

### ETGs differential expression analysis

**Figure 4:**
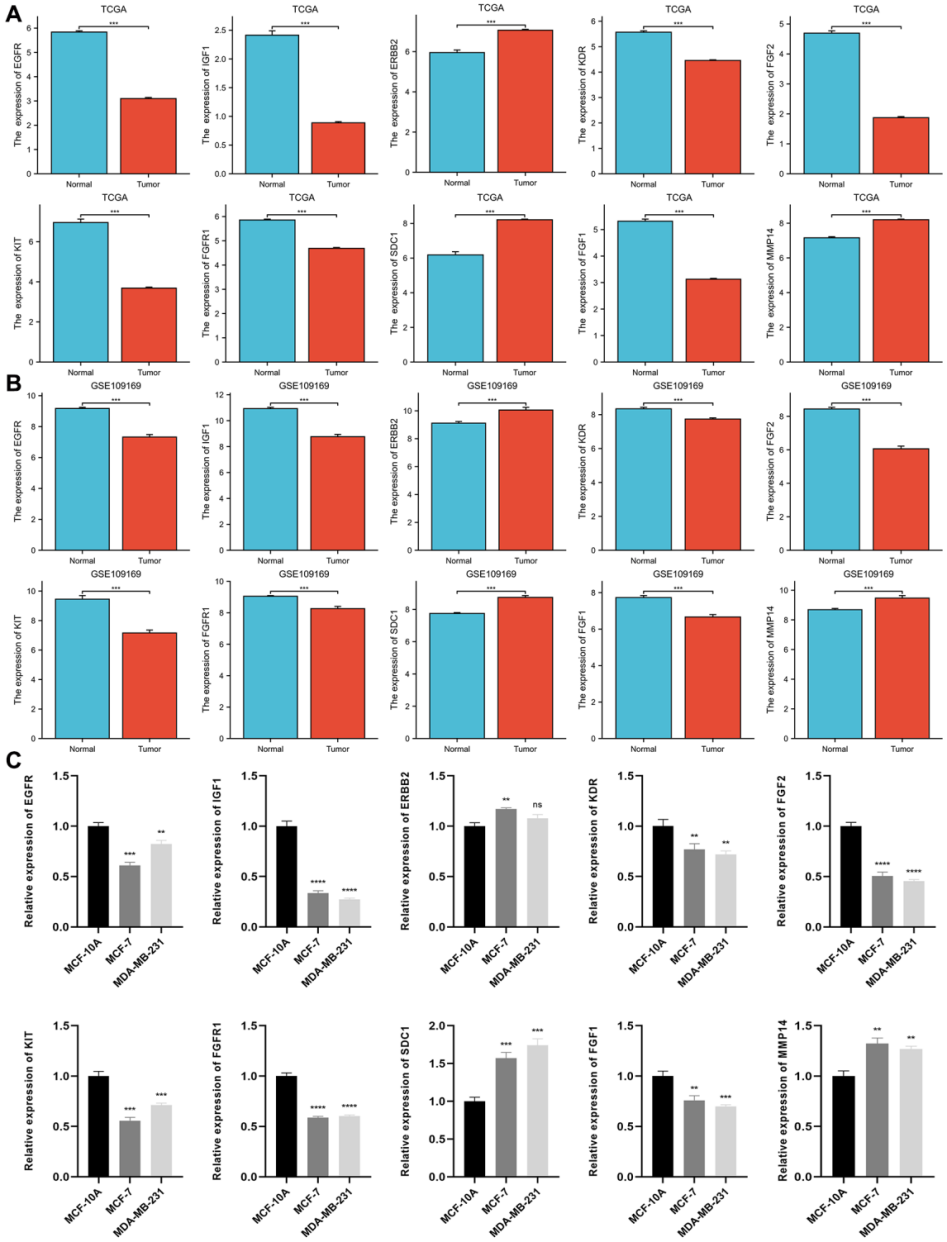
Differential expression of 10 ETGs in BC and normal adjacent tissues based on TCGA database (**A**) and validated by the GSE109169 dataset obtained from the GEO database (**B**) and cell lines (**C**).

**Figure 5:**
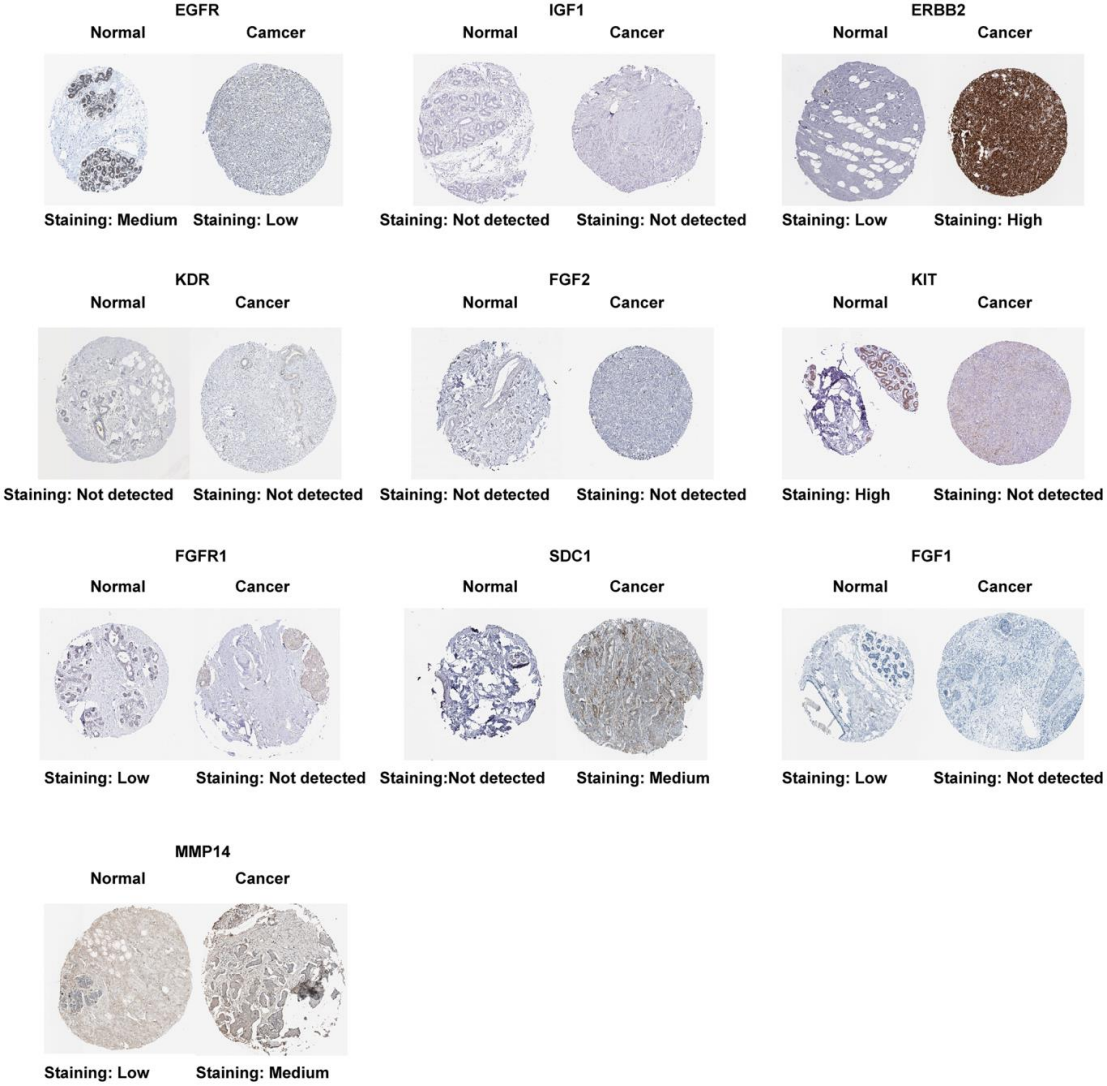
Protein expression of 10 ETGs.

The result of the differential expression indicated that seven out of ten ETGs had reduced expression levels in the tumor group compared to the normal group (Figure 4A). These ETGs were EGFR, IGF1, KDR, FGF2, KIT, FGFR1 and FGF1. In contrast, ERBB2, SDC1, and MMP14 exhibited elevated expression levels in the tumor group (All *p* < 0.05). Furthermore, the GSE109169 dataset was utilized to validate the differential expression of the ETGs, as depicted in Figure 4B. In addition, the analysis of ETGs in cell lines validated the same outcomes (Figure 4C). Figure 5 displays the immunohistochemistry images acquired from the HPA database (https://www.proteinatlas.org/) (Supplementary Table 5) for the analysis of ETGs protein expression. The majority of ETGs showed consistent protein expression with the previous analyses in tissue samples. However, the protein expression of IGF1, KDR and FGF2 were not detected in both BC tissue and normal tissue. Moreover, IHC staining was conducted to confirm the protein expression of three ETGs that were upregulated. We utilized tissue microarrays containing 24 instances of BC tissues and their corresponding neighboring tumor tissues. According to the findings, in 14 out of 24 cases (58.3%) ERBB2 expression was upregulated in BC tissues compared to the corresponding paracancerous tissues, while SDC1 expression was upregulated in 18 out of 24 cases (75.0%). Additionally, 17 out of 24 cases (70.8%) exhibited increased expression. Supplementary Figure 1 contains representative IHC images.

### The diagnostic significance of the upregulated ETGs

**Figure 6:**
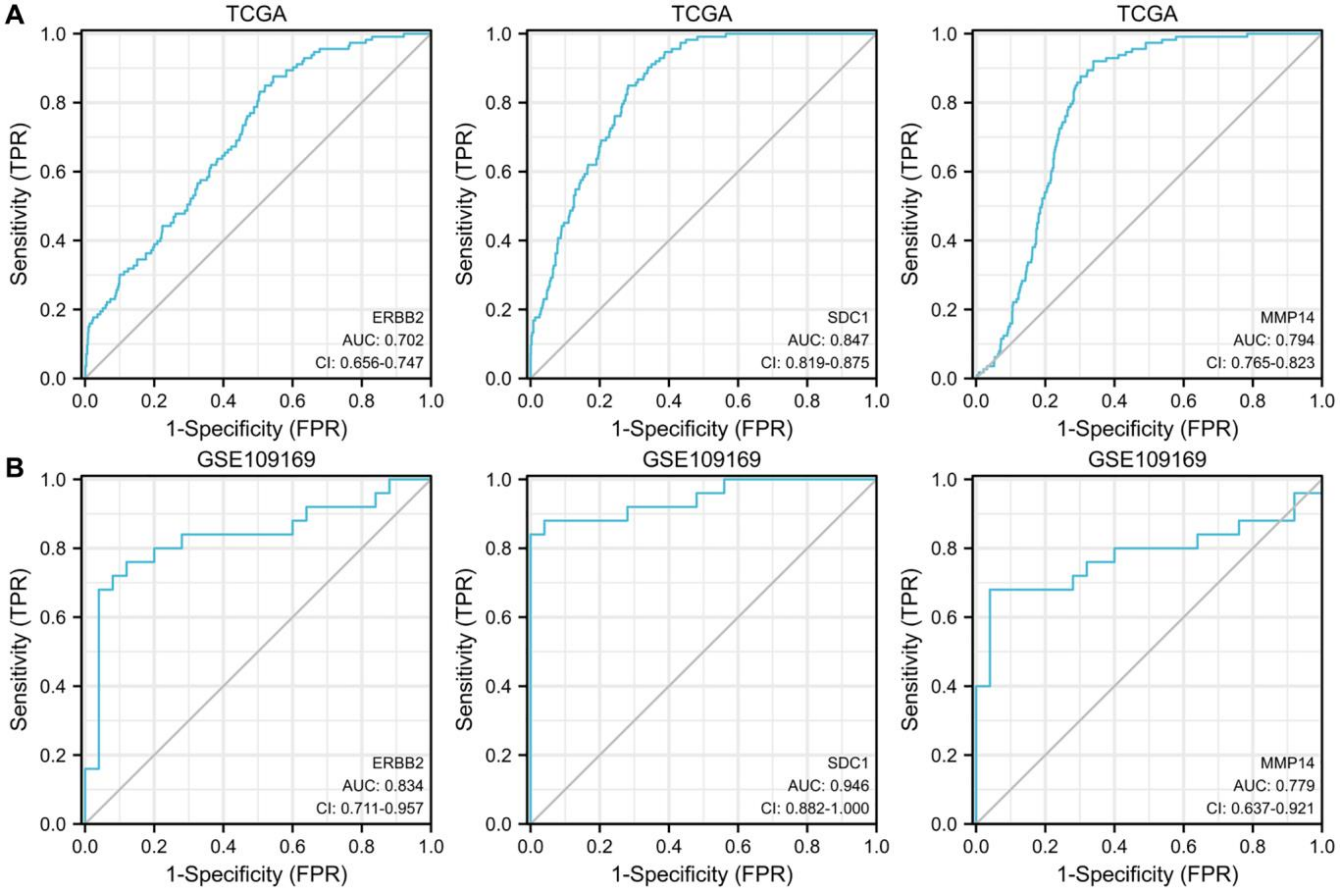
ROC curves show the diagnostic values of 3 upregulated ETGs (**A**) and are validated by the GSE109169 dataset (**B**).

Furthermore, we evaluated the possible diagnostic significance of the upregulated ETGs. The ROC curves for ERBB2, MMP14, and SDC1 are illustrated in Figure 6A. When the threshold value was set at 7.007, the AUC for ERBB2’s ROC curve was 0.702, with a sensitivity of 87.6% and a specificity of 45.6%. When the threshold was set at 7.728, the AUC for MMP14’s ROC curve was 0.794, demonstrating a sensitivity of 92.0% and a specificity of 66.1%. Significantly, SDC1 exhibited the greatest AUC of 0.847 among the ETGs that were upregulated, demonstrating a sensitivity of 85.0% and a specificity of 71.8% when the threshold was established at 7.538. Figure 6B demonstrates the validation of the diagnostic values of the upregulated ETGs using the GSE45666 dataset.

### ETGs clinical significance analysis

**Figure 7:**
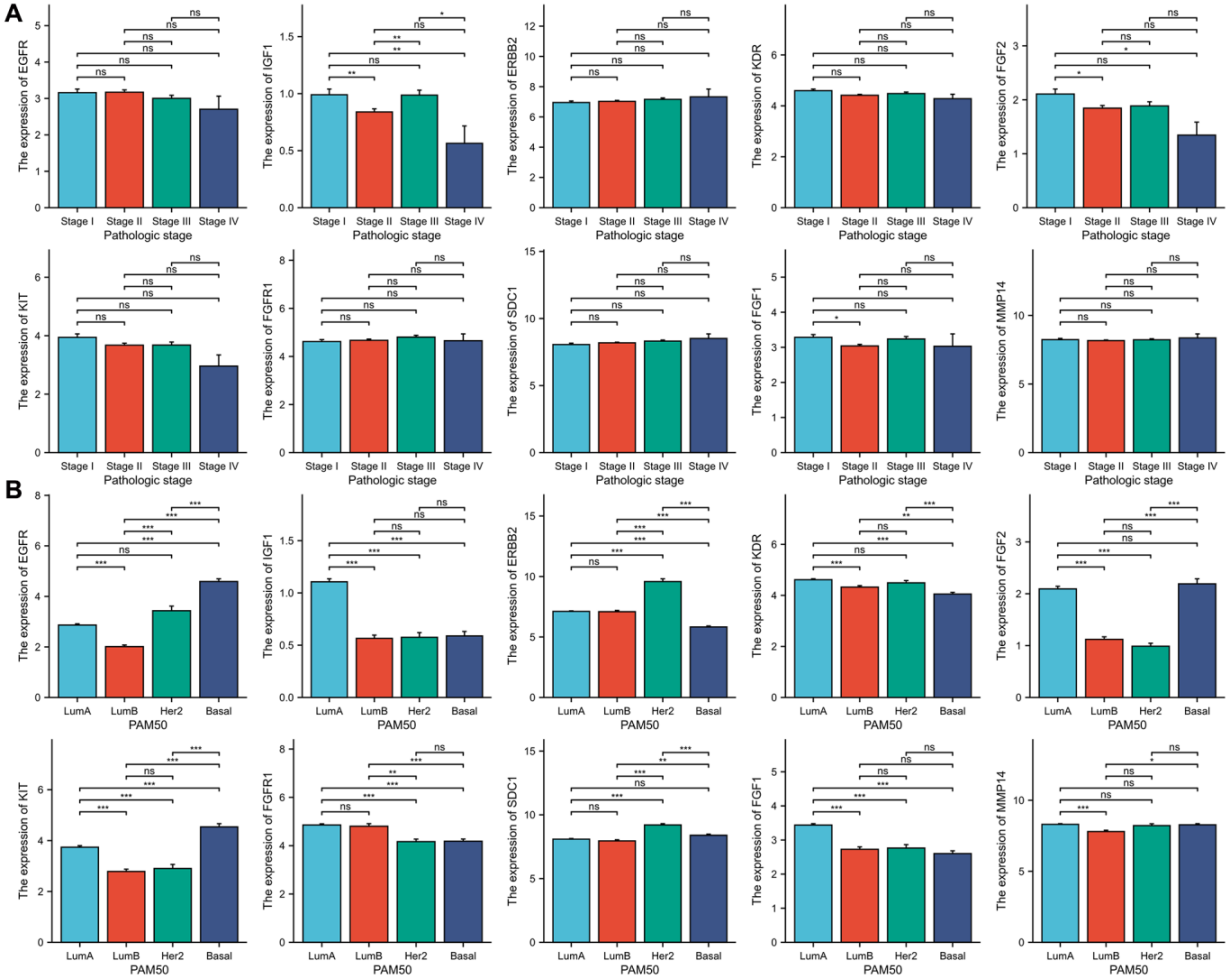
The association between 10 ETGs expression and pathologic stage (**A**) and PAM50 subtype (**B**).

Our study examined the association between ETGs expression and both clinical stages and PAM50 subtypes of BC. As depicted in Figure 7A, in terms of clinical stages, the majority of ETGs exhibited no notable variations in expression across different stages. Nevertheless, the examination in Figure 7B exposed connections between ETGs manifestation and PAM50 subtypes. In particular, the Basal-like subtype exhibited a tendency towards increased expression of EGFR and KIT in comparison to the other subtypes. Conversely, the Basal-like BC exhibited decreased expression of KDR. Luminal A subtype showed increased expression of IGF1 and FGF1. Furthermore, the levels of ERBB2 and SDC1 were elevated in the HER2-enriched BC. In the Luminal subtypes, FGFR1 showed increased expression, while FGF2 showed elevated expression in both Luminal A and Basal-like BC compared to the remaining subtypes.

### Evaluation of the ETGs prognosis

**Figure 8:**
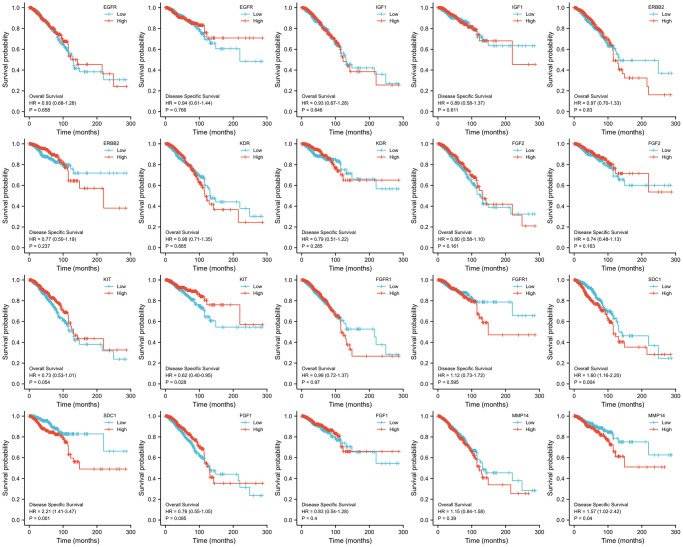
KM survival curves analysis of 10 ETGs.

KM survival curves were generated to analyze the prognosis and the results are presented in Figure 8. It was observed that BC patients who had elevated levels of SDC1 expression experienced poorer DSS (HR = 2.21, *p* = 0.001) and OS (HR = 1.60, *p* = 0.004). Notably, a decrease in KIT expression was linked to a poorer DSS outcome (HR = 0.62, *p* = 0.028), although there was no significant statistical variation in OS. Moreover, increased MMP14 expression was associated with poorer DSS outcomes (HR = 1.57, *p* = 0.040), while no significant correlation was observed with OS.

### Immune infiltration analysis

**Figure 9:**
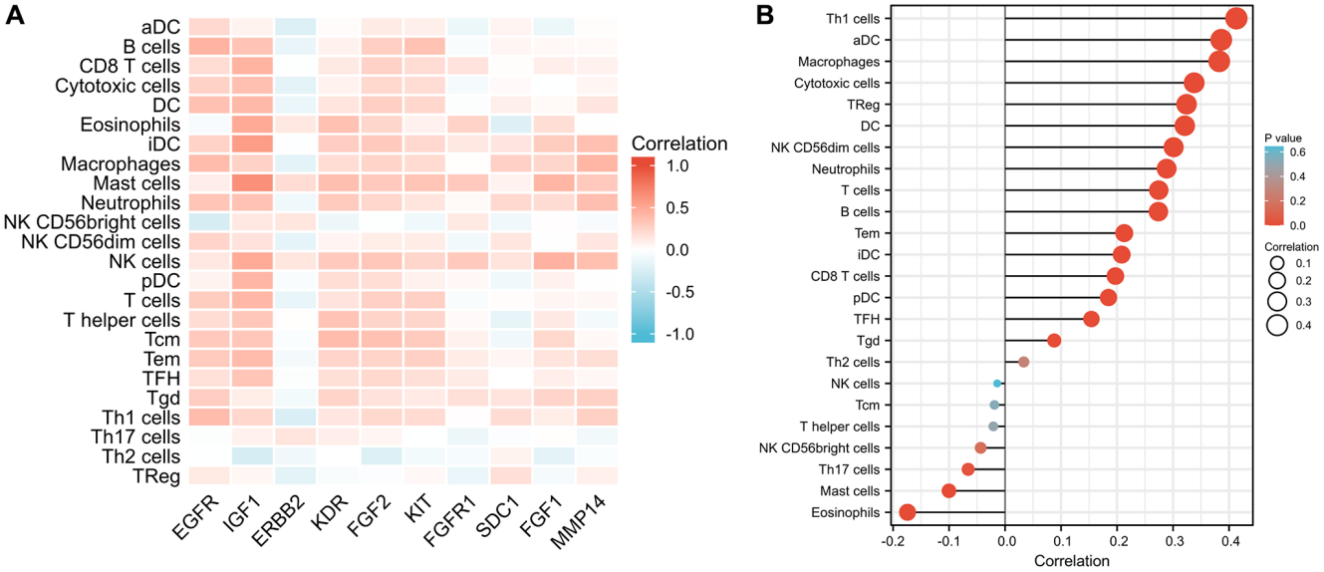
Comparison of infiltration levels in 24 common immune cells between low and high expression groups of 10 ETGs (**A**) and miR-221-3p (**B**).

The results of the immune infiltration analysis are depicted in Figure 9A. We observed that several ETGs, particularly EGFR, IGF1, KDR, FGF2, and KIT, exhibited significant positive correlations with various immune cell infiltrations. Among them, IGF1 showed the strongest positive correlation with CD8+ T cells (r = 0.444), cytotoxic cells (r = 0.366), dendritic cells (DCs) (r = 0.408), eosinophils (r = 0.491), immature DCs (iDCs) (r = 0.571), mast cells (r = 0.464), natural killer (NK) cells (r = 0.486), plasmacytoid DCs (pDCs) (r = 0.425), T cells (r = 0.414), T effector memory (Tem) cells (r = 0.39), and T follicular helper (TFH) cells (r = 0.337) (all *p* < 0.001). On the other hand, a negative correlation between ETG expression and immune infiltration was mainly observed in ERBB2, which exhibited the strongest negative correlation with activated DCs (aDCs) (r = −0.226), B cells (r = −0.140), cytotoxic cells (r = −0.179), DCs (r = −0.129), macrophages (r = −0.174), NK CD56- cells (r = −0.166), T cells (r = −0.150), type 1 Th (Th1) cells (r = −0.243), and regulatory T (Treg) cells (r = −0.180) (all *p* < 0.001), as well as gamma delta T (Tgd) cells (r = −0.079, *p* = 0.008). Moreover, as illustrated in Figure 9B, the miR-221-3p expression exhibited noteworthy positive associations with the majority of immune cell categories, notably Th1 lymphocytes (r = 0.413, *p* < 0.001), aDCs (r = 0.386, *p* < 0.001), and macrophages (r = 0.382, *p* < 0.001). In contrast, the expression of miR-221-3p showed a notable inverse association with eosinophils (r = −0.174, *p* < 0.001) and mast cells (r = −0.100, *p* = 0.001).

### Correlation with the ICGs expression

**Figure 10:**
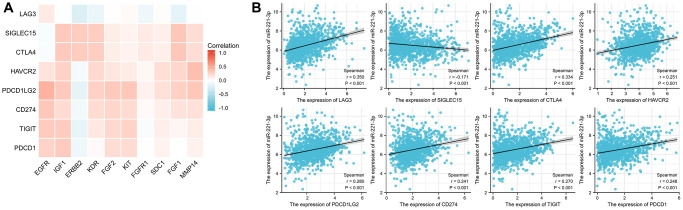
Correlation between ICGs and ETGs expression (**A**), and correlation between ICGs and miR-221-3p expression (**B**).

The findings from Figure 10A indicated that most ETGs controlled by miR-221-3p displayed a positive association with ICGs expression. However, the inverse relationship primarily existed between ERBB2 and ICGs, especially LAG3 (r = −0.229), PDCD1LG2 (r = −0.159), TIGIT (r = −0.155), and PDCD1 (r = −0.109) (all *p* < 0.001). In addition, as depicted in Figure 10B, miR-221-3p displayed a notable association with eight ICGs, out of which it solely demonstrated an inverse correlation with SIGLEC15 (r = −0.170, *p* < 0.001).

### TMB and MSI analysis

**Figure 11:**
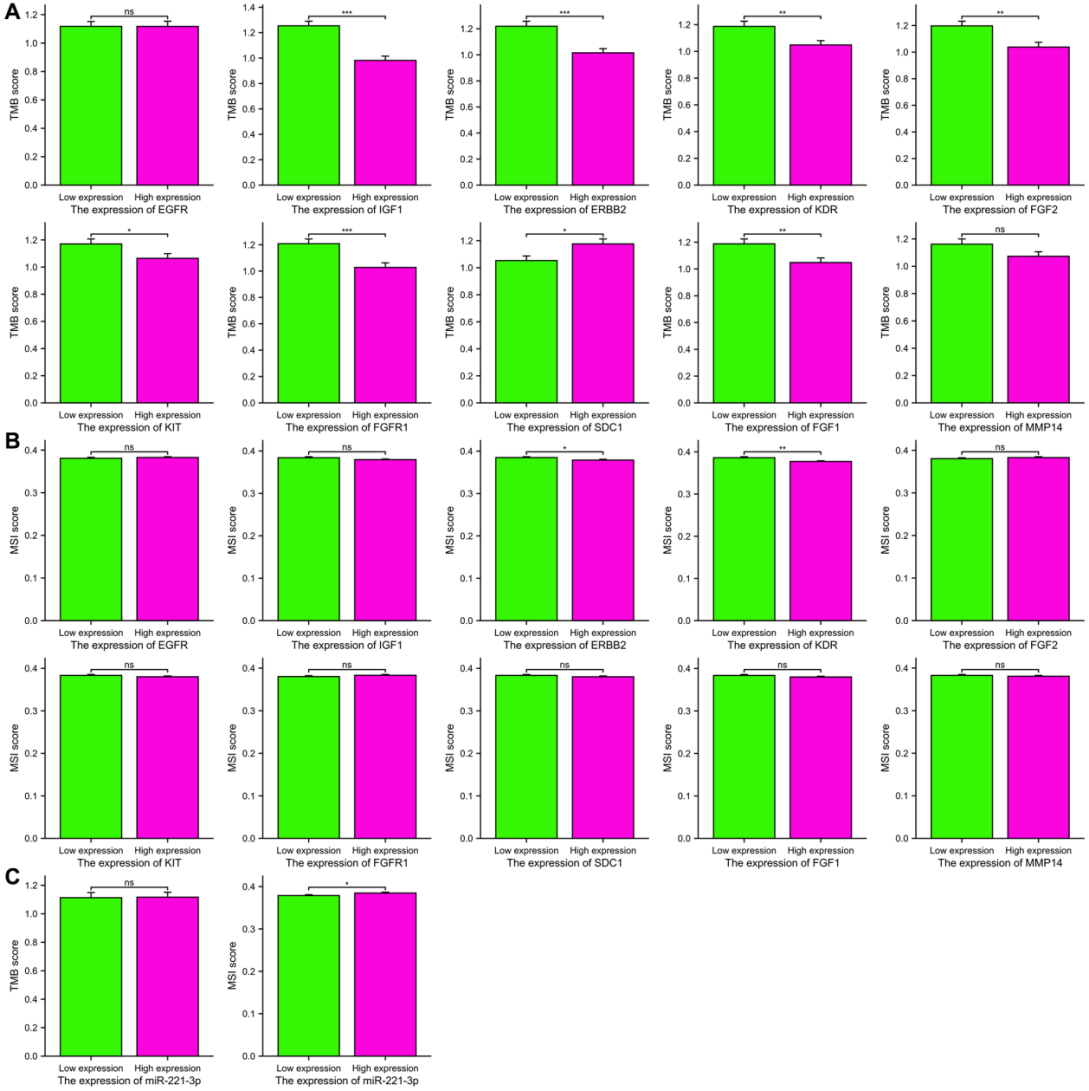
The TMB scores (**A**) and MSI scores (**B**) between the high and low expression groups of 10 ETGs. The TMB and MSI scores between the high and low expression groups of miR-221-3p (**C**).

TMB has become a significant indicator for forecasting the effectiveness of immunotherapy and has been extensively researched in different forms of cancer. Figure 11A demonstrates a positive correlation between higher expression of IGF1 (*p* < 0.001), ERBB2 (*p* < 0.001), KDR (*p* = 0.006), FGF2 (*p* = 0.002), KIT (*p* = 0.035), FGFR1 (*p* < 0.001), and FGF1 (*p* = 0.005) with lower TMB scores. Furthermore, solely elevated SDC1 expression exhibited a correlation with an increased TMB score (*p* = 0.014). Additionally, we examined the correlation between MSI scores and the levels of expression of ETGs, as shown in Figure 11B. The findings from our study indicated that higher MSI scores were linked to decreased expression of ERBB2 and KDR (*p* = 0.036; *p* = 0.002, respectively). Moreover, individuals exhibiting elevated levels of miR-221-3p expression displayed a tendency towards increased MSI scores (*p* = 0.038) as depicted in Figure 11C.

### Stemness analysis

**Figure 12:**
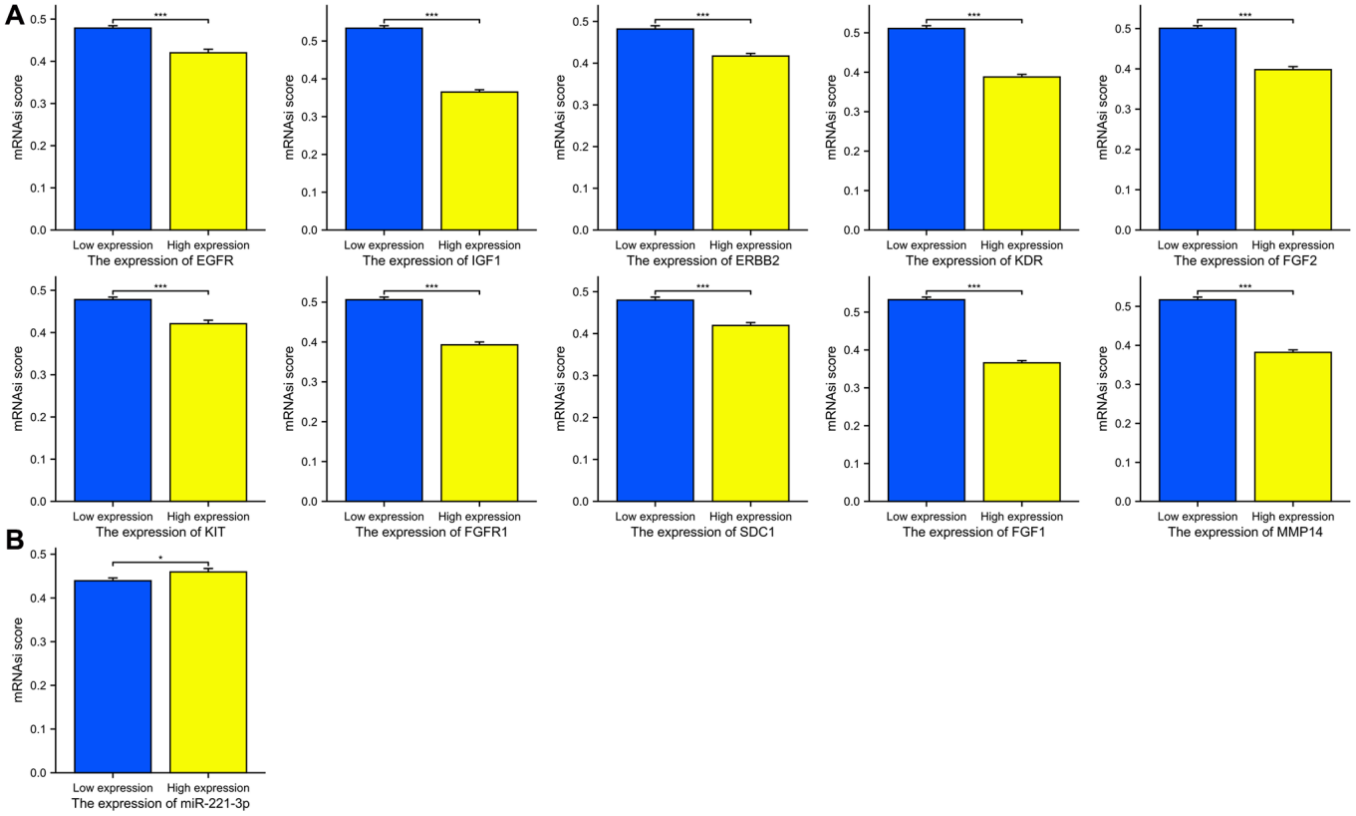
The mRNAsi scores between the high and low expression groups of 10 ETGs (**A**) and miR-221-3p (**B**).

The mRNAsi scores were compared between groups with high expression and low expression. According to Figure 12, there was a significant correlation (*p* < 0.001 for all) between increased expression of ETGs and decreased mRNAsi scores. Conversely, elevated levels of miR-221-3p showed a tendency towards increased mRNAsi scores (*p* = 0.049).

### Drug sensitivity analysis

**Figure 13:**
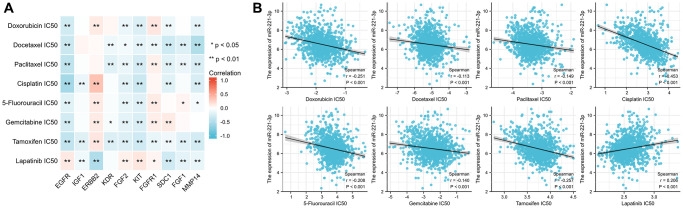
The correlation between the IC50 of 8 drugs and ETGs expression (**A**) and miR-221-3p expression (**B**).

The analysis shown in Figure 13A indicated a majority of ETGs displayed a negative association with the IC50 values. Notably, EGFR exhibited the most robust correlation with IC50 values for doxorubicin (r = −0.297), paclitaxel (r = −0.401), cisplatin (r = −0.560), gemcitabine (r = −0.285), and tamoxifen (r = −0.394) (all *p* < 0.001). Conversely, positive association predominantly existed between the IC50 values and both ERBB2 and FGFR1. Furthermore, Figure 13B revealed a negative correlation between miR-221-3p expression and seven drug IC50 values (all *p* < 0.001), except for lapatinib which showed a positive correlation with miR-221-3p expression (r = 0.206, *p* < 0.001).

### ETGs genetic alteration analysis

**Figure 14:**
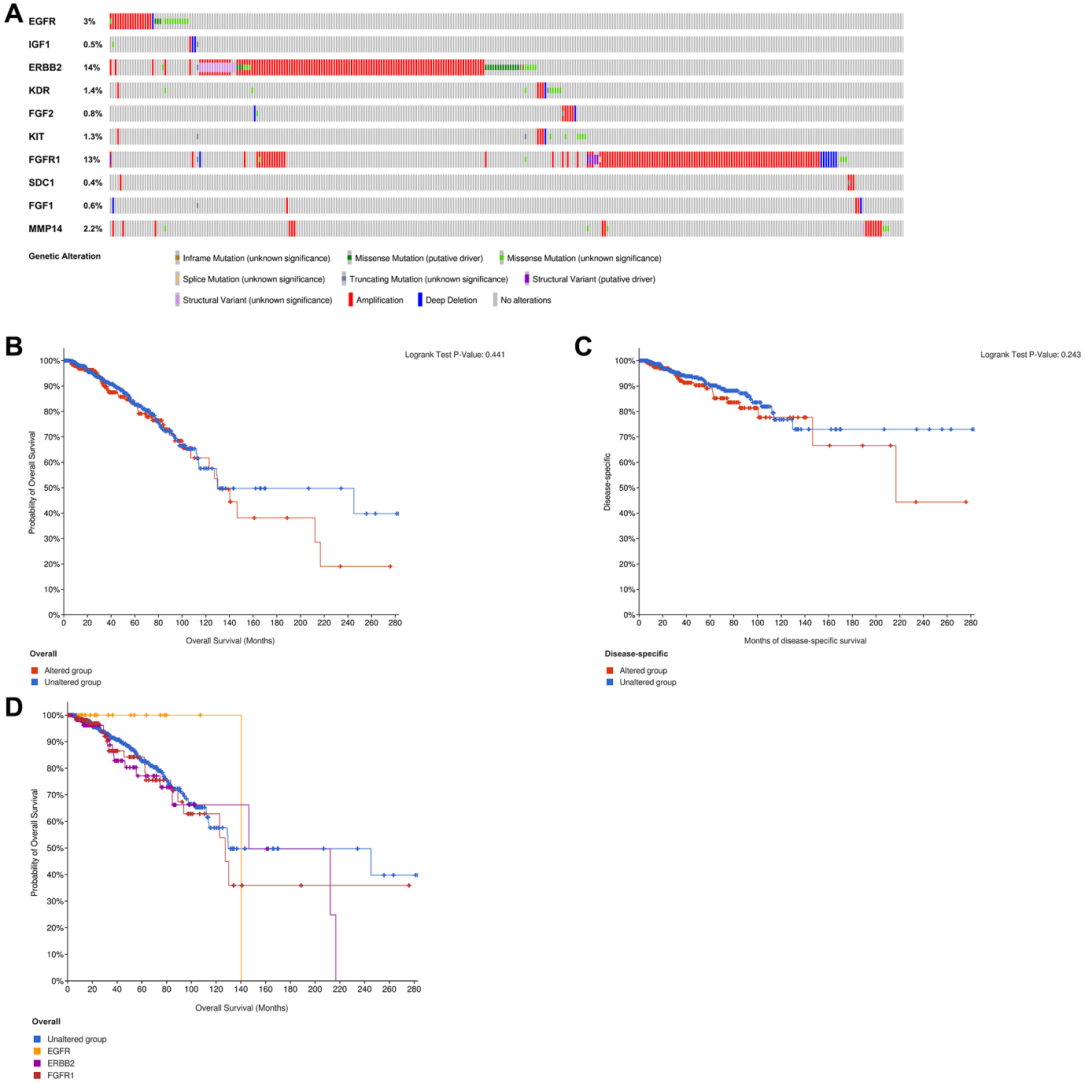
Analysis of genetic alteration of 10 ETGs (**A**). The KM survival curves show the OS and DSS between the ETGs altered group and the unaltered group (**B**, **C**). The KM survival curve shows the OS of the ETGs-unaltered group and the ETGs-altered groups of EGFR, ERBB2 and FGFR1 (**D**).

The examination of genetic alteration of the ETGs depicted in Figure 14A indicated that amplification was the main type of genetic alteration observed in nine ETGs, except for IGF1, which had a genetic alteration rate of only 0.5%. Among the ten ETGs, ERBB2 had the highest genetic alteration rate, reaching 14%. However, there was no notable disparity in OS and DSS between the ETGs-altered group and the unaltered group (Figure 14B, 14C). Furthermore, the OS of the unaltered group and the main ETGs-altered groups (EGFR, ERBB2, and FGFR1) were also analyzed (Figure 14D). The median OS in months (95% CI) for the unaltered group was 129.70, which was longer than that of the FGFR1-altered group (127.33) but shorter than the EGFR (140.28) and ERBB2-altered group (146.50).

## DISCUSSION

Typically, early-stage BC patients have a positive prognosis and a high chance of being cured. Nevertheless, the poor prognosis of metastatic BC poses a considerable public health obstacle. Over the past few years, there has been an increasing fascination with the phenomenon of EMT as a pivotal process implicated in the spread of tumors [5]. The progression of BC is influenced by the participation of multiple miRNAs in the regulation of EMT. These miRNAs can have tumor-suppressing or tumor-promoting effects and may serve as potential therapeutic targets [11, 26]. It has been demonstrated that miR-221-3p is a regulator of EMT in BC [14, 15, 27]. Using bioinformatic analyses, we identified ten genes associated with EMT that are targeted by miR-221-3p. Additionally, we delved into the potential mechanisms that regulate these genes.

In this study, miR-221-3p expression was observed to be reduced when compared to the adjacent normal tissues. The validation of this finding was additionally confirmed in the MCF-7 cell line through qRT-PCR. However, prior research has consistently indicated elevated levels of miR-221-3p expression in blood and tissue samples from individuals with BC [28, 29]. These discrepancies could be attributed to small sample sizes and regional differences in patient selection, mainly in Asian populations, which may have contributed to inconsistent results. Our research uncovered a noteworthy increase in the expression of miR-221-3p in MDA-MB-231 cells. Furthermore, the analysis of PAM50 subtypes indicated a considerably higher expression in the Basal-like subtype when compared to other subtypes of BC and normal tissues. These findings align with previous studies [14, 15, 30]. The ROC analysis indicated that miR-221-3p could potentially function as a distinctive biomarker for Basal-like BC. Moreover, the miR-221-3p’s clinical significance suggests that its elevated levels were inversely linked to the status of ER, PR, and HER2. Studies have shown that the excessive presence of miR-221-3p hinders the translation of ER. However, ER has the ability to repress the expression of miR-221-3p through the recruitment of nuclear receptor corepressor and thyroid hormone receptor [31]. The specific relationship between miR-221-3p and PR or HER2 remains unclear. Furthermore, our investigation uncovered the correlation between miR-221-3p expression and the infiltration of immune cells, along with ICGs, indicating its potential involvement in the regulation of the immune microenvironment in BC. Moreover, we discovered a direct association between the miR-221-3p expression and mRNAsi scores, suggesting that individuals with elevated levels of miR-221-3p in BC exhibited reduced levels of differentiation and increased cellular stemness.

The pathway enrichment analysis in our research indicated that miR-221-3p has the potential to control the process of EMT through the MAPK signaling pathway. Abnormal activation of the p38 MAPK signaling pathway in BC cells has been demonstrated to promote EMT, one of the three primary components of the MAPK signaling pathway [32]. For our research, we identified ten key target genes related to EMT of miR-221-3p for subsequent analysis.

The epidermal growth factor receptor (EGFR), a transmembrane protein, is essential for controlling cellular processes like growth, development, and viability. MiR-221-3p has been recognized in recent research as a regulator of the EGFR signaling pathway that enhances EMT [33, 34]. Based on the findings of this research, miR-221-3p exhibits a strong association with EGFR, which emerging as the top core ETG, and there is also a positive correlation in their expression levels. The results indicate that the combination of miR-221-3p and EGFR activation may collaborate in enhancing EMT in BC through the establishment of a beneficial cycle. According to reports, EGFR overexpression is found in 15-30% of cases of BC, and overexpression is observed in at least 50% of Basal-like BC [34, 35]. Furthermore, we noticed a substantial increase in the expression of EGFR in Basal-like BC in comparison to other subtypes, suggesting a potential correlation between its overabundance and the invasion of BC. Furthermore, our findings indicate a correlation between the elevated levels of EGFR and the presence of immune cell infiltrates and ICGs. This implies that individuals diagnosed with BC who exhibit high EGFR expression might experience greater advantages from immune checkpoint inhibitor therapy. In recent times, the treatment of Basal-like BC [36] has been effectively demonstrated by the emerging therapy of chimeric antigen receptor T-cell that specifically targets EGFR. EGFR seems to be a promising therapeutic focus for treating Basal-like BC, as indicated by these findings.

Insulin-like growth factor 1 (IGF1) is crucial in the metabolic function of hepatocytes and the overall metabolism of the body [37]. Previous experimental findings demonstrated that IGF1 facilitates the activation of EMT through the MAPK and PI3K/AKT pathways, consequently promoting the metastasis of BC cells [38]. This study revealed that IGF1 exhibited the strongest positive association with the majority of immune cells, including CD8+ T cells, DCs, NK cells, and T cells. This suggests that IGF1 signaling molecules could potentially attract immune cells within the tumor microenvironment. Nevertheless, there is a scarcity of prior research on the impact of IGF1 signaling on immune cells in BC. Consequently, further experiments are necessary to uncover its function to anti-tumor immunity.

Erb-b2 receptor tyrosine kinase 2 (ERBB2), also known as HER2, is a receptor tyrosine kinase belonging to the EGFR family. It is amplified and overexpressed in over 20% of BC cases, leading to an unfavorable prognosis [39]. Multiple pieces of evidence indicate that ERBB2 has the ability to trigger EMT in BC cells by engaging with various pathways associated with stemness, leading to the development of resistance to trastuzumab [40]. Moreover, the excessive expression of ERBB2 in breast epithelial cells has the ability to trigger EMT and enhance oncogenic capacity, which can be suppressed by the concurrent presence of EGFR [41]. In our present investigation, we discovered an inverse association between the expression of ERBB2 and numerous infiltrations of immune cells, indicating that ERBB2 may also play a role in suppressing the immune response in BC. The presence of ERBB2 was also discovered to have a positive correlation with the IC50 values of chemotherapeutic medications, indicating that ERBB2 signaling could potentially be involved in the development of resistance to chemotherapy.

Kinase inserts domain receptor (KDR), also called VEGFR2, is a receptor for vascular endothelial growth factor and plays a crucial role in regulating angiogenesis in BC. Overexpression of KDR is linked to the aggressive advancement of BC [42, 43]. Furthermore, there have been reports indicating a correlation between elevated KDR expression and the heightened expression of proteins associated with EMT in BC. However, the precise mechanism remains unknown [43]. Additionally, there were reports indicating that elevated KDR expression can facilitate the conversion of BC from Basal-like to Luminal phenotype and improve the responsiveness to Tamoxifen therapy, which is linked to a favorable prognosis [44]. In this study, we observed comparable results where the luminal-like subtype exhibited elevated levels of KDR expression compared to the Basal-like subtype. Furthermore, heightened KDR expression was linked to reduced responsiveness to various drugs, including Tamoxifen. The role of KDR in BC remains controversial and deserves further exploration.

As a member of the fibroblast growth factor (FGF) family, FGF2 is a growth factor derived from cancer-associated fibroblasts that stimulate BC cell proliferation and migration [45, 46]. Interestingly, another study found that FGF2 can reverse the TGF-β signaling pathway to suppress the growth and migration of BC cells [47]. Although FGF2 has been identified as an EMT activator in the progression of cancers [48, 49], studies about its EMT-related regulatory mechanisms in BC are limited. Our finding reveals for the first time that FGF2 might recruit immune cells and enhance the drug sensitivity to exert an anti-tumor effect in BC.

The KIT proto-oncogene receptor tyrosine kinase (KIT) gene codes for the cluster of differentiation 117, a receptor tyrosine kinase responsible for controlling cellular growth and viability [50]. The expression and function of KIT in BC have been a topic of considerable controversy. On the one hand, prior research has indicated that the occurrence of KIT expression in patients with Basal-like BC is higher compared to patients with other subtypes, and an elevated KIT expression level is linked to the advancement of tumors [51, 52]. In this research, we discovered a similar result indicating that the level of KIT expression is higher in Basal-like BC compared to other subcategories. Conversely, certain studies have indicated that the absence of KIT is detected while breast cancer is progressing and is associated with the occurrence of malignancy [53, 54]. In line with these results, our study revealed a decrease in KIT expression in BC tissues compared to normal tissues. Additionally, patients with elevated KIT expression had a longer DSS period. Furthermore, it was discovered that increased KIT expression is associated with elevated immune infiltration and expression of ICGs, as well as decreased drug sensitivity and stemness. This suggests that BC patients with higher levels of KIT expression are more likely to respond well to treatment and have a favorable prognosis. The possible reason for the controversial role of KIT in BC could be explained by tissue specificity [53]. KIT might mainly function as a tumor suppressor gene in BC but promote the malignant transformation in the Basal-like subtype, which deserves further in-depth studies.

As a member of the fibroblast growth factor receptor (FGFR) family, FGFR1 shows a high affinity for FGF2 to stimulate the growth and progression of BC cells [45]. FGFR1 was recently demonstrated as an EMT marker and its interaction with β3 integrin is required to the FGF2-facilitate metastatic outgrowth in BC [55]. The co-expression between FGFR1 and FGF2 was also found in this study. Additionally, it was reported that FGFR1 amplification mainly existed in the Luminal subtype and is related to adverse prognosis [56, 57]. We found similar outcomes in this study, the amplification rate of FGFR1 in the TCGA dataset was up to 13%, and the median months overall of the FGFR1 altered BC patients was 127.33, shorter than the unaltered group. Hence, it is imperative to conduct additional research on FGFR1 as a plausible target for therapeutic intervention in BC.

Syndecan-1 (SDC1), a heparan sulfate proteoglycan, is part of the syndecan family and is crucial in the advancement of cancer [58]. In this study, we discovered that the SDC1 demonstrates a greater level of expression in BC, particularly in the HER2-enriched subtype. Additionally, BC patients with elevated SDC1 levels are more likely to experience a negative prognosis. These findings align with previous investigations [59–61]. Furthermore, the AUC value of the diagnostic ROC curve is 0.847, indicating that SDC1 has the potential to be a biomarker for the diagnosis of BC, with a sensitivity of 85.0% and specificity of 71.8%.

FGF1 is part of the FGF family, known for its role in promoting angiogenesis [62]. It was observed that FGF1 expression in mammary epithelial cells can augment EMT induced by TGF-β1 [63], which might be crucial to the occurrence of BC. In line with a prior investigation, our findings indicate that the levels of FGF1 in BC tissues are comparatively reduced compared to those in normal breast tissues, implying that FGF1 primarily functions as a differentiating agent in normal tissues rather than a factor promoting growth. Nevertheless, the involvement of FGF1 expression in BC has been documented in the invasion and spread of BC. Additionally, the antibody scFv1C9, specific to FGF1, has demonstrated its ability to decrease the density of microvessels in BC tissues and hinder the lung metastasis of BC [64–66]. These findings highlight the potential of IGF1 as a target for effective BC treatment.

As a member of the matrix metalloproteinase (MMP) family, MMP14 is overexpressed in BC tissues and participates in the pathogenesis and metastasis of BC [67]. Correlative evidence suggests that high MMP14 expression in cancers is significantly correlated with adverse prognosis [68–70], and it was observed that BC patients with high MMP14 expression have a shorter DSS. It was the first time we found that MMP14 exhibited a high correlation with immune cells, ICGs expression, and drug sensitivity in BC. Additionally, by the ROC analysis, we found that MMP14 might be a diagnostic biomarker for BC. It has been demonstrated that the MMP family is crucial in regulating the EMT process in cancers [71, 72], however, the EMT-associated mechanism of MMP14 involved in BC has not been reported, we supposed that miR-221-3p might upregulate the MMP14 expression in BC to promote EMT, which deserves our further study.

Further validation is necessary for the present study, as it primarily relied on data acquired from the TCGA database. To overcome these limitations, it is recommended to conduct the luciferase reporter assay as an initial measure in order to confirm the correlation between miR-221-3p and its ETGs. Secondly, further research should be conducted on the EMT-regulated mechanisms associated with ETGs. Moreover, it is imperative to explore the potential of employing ETGs as targets for therapy in the upcoming time. The results of this study have implications for future research on the EMT mechanism of BC, potentially offering effective treatment approaches for individuals afflicted by the condition.

## CONCLUSION

To summarize, our research revealed that miR-221-3p is upregulated in Basal-like BC, functioning as a distinctive indicator for differentiating it from other subtypes of BC. Ten core ETGs of miR-221-3p were identified, and SDC1 and MMP14 could potentially function as valuable indicators for the diagnosis of BC and the prediction of unfavorable prognosis. The comprehensive analysis of these ten ETGs indicates their possible involvement in the tumor microenvironment during the development of BC. These findings highlight the promising therapeutic targets for BC patients.

## Supplementary Materials

Supplementary Figure 1

Supplementary Table 1

Supplementary Table 2

Supplementary Table 3

Supplementary Tables 4 and 5
