# Supplementary material for: Clinical significance and immune characteristics analysis of miR-221-3p and its key target genes related to epithelial-mesenchymal transition in breast cancer
Source: Aging (Albany NY). 2024 Jan 6;16(1):322–47. doi: 10.18632/aging.205370 (PMC10817385; doi:10.18632/aging.205370)
Supplement: Supplementary Figure 1 [file aging-16-205370-s001.pdf]

## SUPPLEMENTARY FIGURE

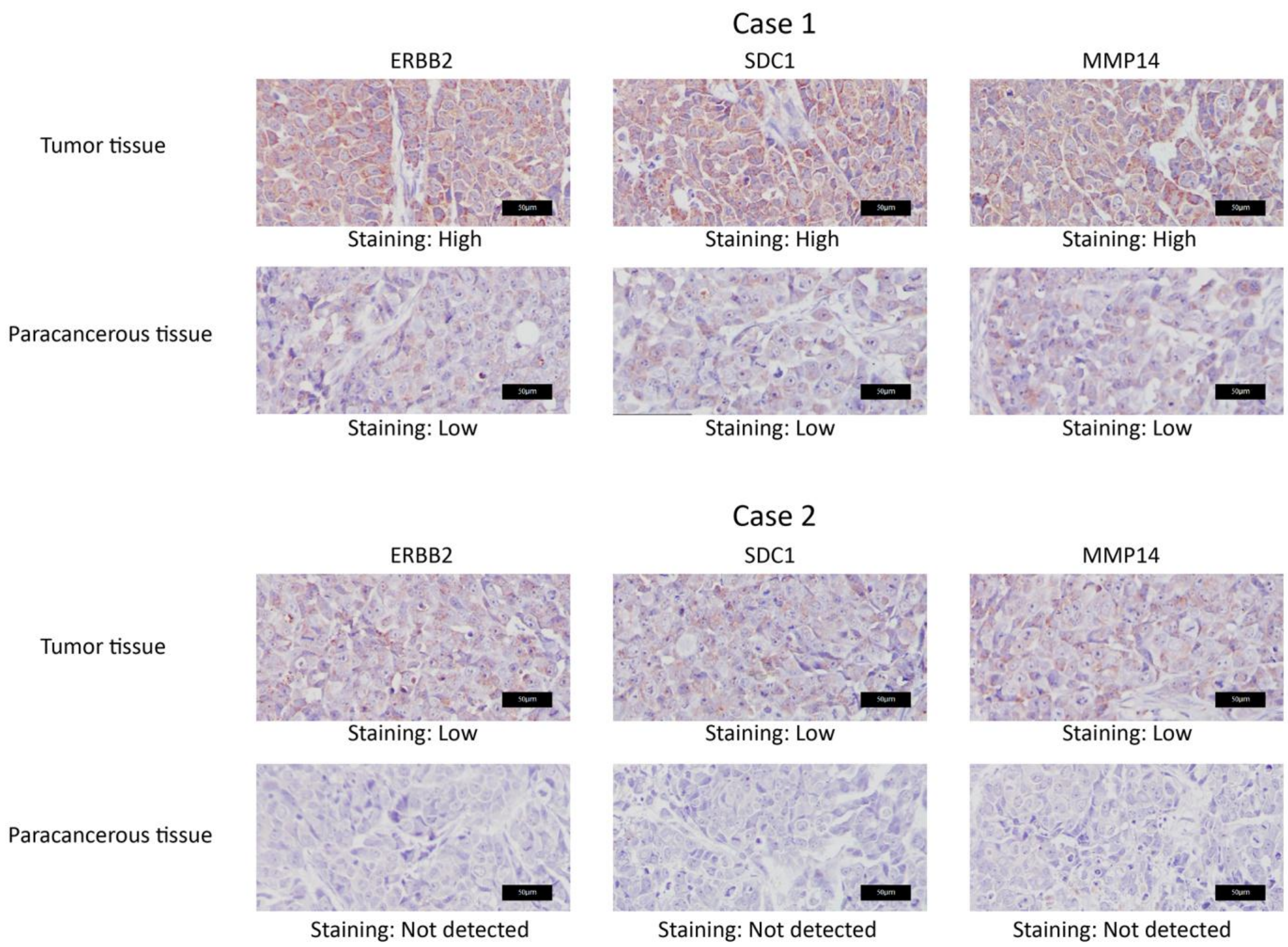


**Supplementary Figure 1. Representative images of 3 upregulated ETGs in BC tissues and their matched paracancerous tissues. Original magnifications 200×.**
