# Supplementary material for: Clinical significance and immune characteristics analysis of miR-221-3p and its key target genes related to epithelial-mesenchymal transition in breast cancer
Source: Aging (Albany NY). 2024 Jan 6;16(1):322–47. doi: 10.18632/aging.205370 (PMC10817385; doi:10.18632/aging.205370)
Supplement: Supplementary Tables 4 and 5 [file aging-16-205370-s005.pdf]

SUPPLEMENTARY TABLES

Supplementary Table 4. GO and KEGG pathway enrichment analysis of 35 ETGs of miR-221-3p.

Ontology	ID	Description	Gene Ratio	BgRatio	*p*value	*p*.adjust	*q*value	geneID	Count
BP	GO:0050679	positive regulation of epithelial cell proliferation	10/35	206/18670	3.10e-12	5.84e-09	3.35e-09	EGFR/ERBB2/FGF1/FGF2/FGFR1/IGF1/KDR/PRKCA/CYP7B1/ESRP2	10
BP	GO:0050673	epithelial cell proliferation	12/35	434/18670	1.10e-11	1.04e-08	5.94e-09	EGFR/ERBB2/FGF1/FGF2/FGFR1/IGF1/KDR/KIT/MMP14/PRKCA/CYP7B1/ESRP2	12
BP	GO:0061138	morphogenesis of a branching epithelium	9/35	182/18670	3.70e-11	1.84e-08	1.05e-08	FGF1/FGF2/FGFR1/MMP14/TGFBR2/SEMA3E/SPRY1/LEF1/ESRP2	9
CC	GO:0009925	basal plasma membrane	2/35	34/19717	0.002	0.067	0.057	EGFR/ERBB2	2
CC	GO:0045121	membrane raft	4/35	315/19717	0.002	0.067	0.057	EGFR/KDR/TGFBR2/CAVIN1	4
CC	GO:0098857	membrane microdomain	4/35	316/19717	0.002	0.067	0.057	EGFR/KDR/TGFBR2/CAVIN1	4
MF	GO:0019199	transmembrane receptor protein kinase activity	8/35	79/17697	2.34e-12	2.81e-10	1.82e-10	EGFR/ERBB2/FGFR1/KDR/KIT/NTRK3/TGFBR2/CRIM1	8
MF	GO:0004714	transmembrane receptor protein tyrosine kinase activity	7/35	62/17697	2.84e-11	1.71e-09	1.11e-09	EGFR/ERBB2/FGFR1/KDR/KIT/NTRK3/CRIM1	7
MF	GO:0005178	integrin binding	8/35	132/17697	1.54e-10	6.15e-09	3.99e-09	EGFR/FGF1/FGF2/IGF1/KDR/MMP14/PRKCA/FBLN5	8
KEGG	hsa04010	MAPK signaling pathway	11/22	294/8076	6.11e-11	7.88e-09	5.27e-09	EGFR/ERBB2/FGF1/FGF2/FGFR1/IGF1/KDR/KIT/PRKCA/TGFBR2/PDGFD	11
KEGG	hsa01521	EGFR tyrosine kinase inhibitor resistance	7/22	79/8076	9.91e-10	5.48e-08	3.67e-08	EGFR/ERBB2/FGF2/IGF1/KDR/PRKCA/PDGFD	7
KEGG	hsa05205	Proteoglycans in cancer	9/22	205/8076	1.37e-09	5.48e-08	3.67e-08	EGFR/ERBB2/FGF2/FGFR1/IGF1/KDR/PRKCA/SDC1/TWIST2	9

Supplementary Table 5. URL that links directly to the HPA database for obtaining the IHC images and information.

Gene	Tissue type	URL
EGFR	normal tissue	https://images.proteinatlas.org/18530/41191_B_2_4.jpg
EGFR	breast cancer tissue	https://images.proteinatlas.org/18530/41188_A_6_7.jpg
IGF1	normal tissue	https://images.proteinatlas.org/48946/110992_B_2_4.jpg
IGF1	breast cancer tissue	https://images.proteinatlas.org/48946/110989_A_6_8.jpg
ERBB2	normal tissue	https://images.proteinatlas.org/1383/4682_B_2_4.jpg
ERBB2	breast cancer tissue	https://images.proteinatlas.org/1383/4496_A_5_6.jpg
KDR	normal tissue	https://images.proteinatlas.org/4028/11467_B_2_4.jpg
KDR	breast cancer tissue	https://images.proteinatlas.org/4028/12048_A_4_1.jpg
FGF2	normal tissue	https://images.proteinatlas.org/65502/167321_B_1_4.jpg

FGF2	breast cancer tissue	https://images.proteinatlas.org/125/2185_A_5_5.jpg
KIT	normal tissue	https://images.proteinatlas.org/72867/156398_B_2_4.jpg
KIT	breast cancer tissue	https://images.proteinatlas.org/72867/156394_A_5_3.jpg
FGFR1	normal tissue	https://images.proteinatlas.org/56402/136145_B_2_4.jpg
FGFR1	breast cancer tissue	https://images.proteinatlas.org/56402/136148_A_4_8.jpg
SDC1	normal tissue	https://images.proteinatlas.org/67477/168250_B_1_4.jpg
SDC1	breast cancer tissue	https://images.proteinatlas.org/6185/40535_A_5_4.jpg
FGF1	normal tissue	https://images.proteinatlas.org/17519/39215_B_2_4.jpg
FGF1	breast cancer tissue	https://images.proteinatlas.org/17519/39212_A_5_2.jpg
MMP14	normal tissue	https://images.proteinatlas.org/9918/24805_B_1_4.jpg
MMP14	breast cancer tissue	https://images.proteinatlas.org/9918/24802_A_6_2.jpg
